# Exploring or Avoiding Novel Food Resources? The Novelty Conflict in an Invasive Bird

**DOI:** 10.1371/journal.pone.0019535

**Published:** 2011-05-18

**Authors:** Daniel Sol, Andrea S. Griffin, Ignasi Bartomeus, Hayley Boyce

**Affiliations:** 1 Centre for Ecological Research and Forestry Applications (CREAF) Autonomous University of Barcelona, Bellaterra, Catalonia, Spain; 2 Center for Advanced Studies of Blanes (CEAB), Spanish National Research Council (CSIC), Blanes, Spain; 3 School of Psychology, University of Newcastle, Callaghan, Australia; University of Lethbridge, Canada

## Abstract

For an animal invading a novel region, the ability to develop new behaviors should facilitate the use of novel food resources and hence increase its survival in the new environment. However, the need to explore new resources may entail costs such as exposing the animal to unfamiliar predators. These two opposing forces result in an exploration-avoidance conflict, which can be expected to interfere with the acquisition of new resources. However, its consequences should be less dramatic in highly urbanized environments where new food opportunities are common and predation risk is low. We tested this hypothesis experimentally by presenting three foraging tasks to introduced common mynas (*Acridotheres tristis*) from environments with low and high urbanization levels from Australia. Individuals from the highly urbanized environments, where mynas are both more opportunistic when foraging and less fearful to predators, resolved a technical task faster than those from less urbanized environments. These differences did not reflect innovative ‘personalities’ and were not confounded by sex, morphology or motivational state. Rather, the principal factors underlying differences in mynas' problem-solving ability were neophobic-neophilic responses, which varied across habitats. Thus, mynas seem to modulate their problem-solving ability according to the benefits and costs of innovating in their particular habitat, which may help us understand the great success of the species in highly urbanized environments.

## Introduction

When exposed to a novel environment, animals are confronted with a variety of new ecological challenges. The ability to cope with such challenges may make the difference between survival and death. At the forefront of these challenges is the need to acquire food supplies. Invaders are likely to be confronted more often with novel foods than familiar ones, so they run the risk of starvation if they are unable to adopt new foraging opportunities. Thus, it is easy to imagine that a species that readily tastes new foods and/or develops novel foraging techniques is more likely to survive and to reproduce in a novel environment than a more stereotyped species that persists with behaviors that were adaptive in its area of origin. These considerations have led to the hypothesis that the success of animals in new environments depends, at least in part, on their behavioral innovation ability [1], [2], a possibility that is supported by comparative analyses of human-aimed introductions of birds and mammals [3], [4].

However, whether or not an individual adopts a new feeding opportunity does not only depend on its innovation ability, but also on its emotional response to novelty challenges [5], [6]. Emotional responses, also called coping styles or personality traits [7], encompass two conflicting forces: the need to approach and to explore new resource opportunities on the one hand, and the need to avoid unnecessary risks, on the other. For an invader, exploring and eating new foods can be dangerous, as food may contain poisons and/or can expose individuals to unfamiliar enemies. But if an invader consistently avoids exploring unfamiliar feeding opportunities, it might have difficulty finding enough food. It follows that emotional responses can either facilitate or interfere with innovation depending on whether an individual opts for approach or avoidance.

**Figure 1:**
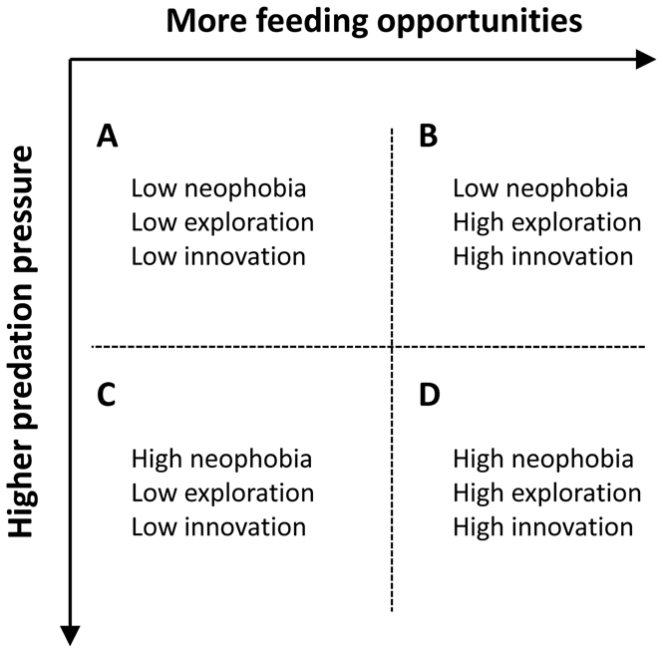
Adaptation of the Two-Factor model proposed by Greenberg & Mettke-Hoffmann [**6**] to describe the interplay between neophobia, exploration and innovation as a function of the ecological context.

The balance between approach and avoidance is expected to differ depending on prevailing ecological conditions ([5], [6]; see Fig. 1, scenarios A–D). In environments where encounters with unfamiliar resources are uncommon and risks associated with native predators are high, invaders should typically lean towards avoidance (Fig. 1, scenario B). In contrast, in environments where exposure to novel feeding opportunities is commonplace and risks associated with native predators are low, invaders should generally favor approach and exploration of novel resources over avoidance (Fig. 1, scenario C). Assuming that exploration pave the way for innovation, it follows that the innovative abilities of an invader will be more or less hindered by the outcome of approach-avoidance conflict depending on the ecological characteristics of the environment to which the invader has been introduced.

Here, we report a “common arena” experiment aimed to determine whether individuals from different environments vary in their innovative abilities, and to what extent differences in the way they weight approach over avoidance underpins differences in innovation. To this end, we used the highly invasive common myna (*Acridotheres tristis*, alias Indian mynah) and compared the behavior of birds from environments with low- and high-levels of urbanization. The rationale for this comparison is that birds from highly urbanized areas should be inclined to favor approach and exploration over avoidance as in these environments predators tend to be scarcer and survival depends largely on the ability to exploit human-derived resources that differ from those found naturally [8]. This idea is supported by evidence that birds from urbanized environments adopt novel feeding opportunities more readily [9], [10], [11] and tend to show shorter flight distances to approaching predators [10] than birds from less urbanized environments, a pattern also found in free-ranging common mynas (see Text S1, Figure S1). As a consequence, in highly urbanized environments the outcome of the approach-avoidance conflict should interfere less with the acquisition of new resources, making these environments potentially easier to invade than less urbanized environments. Although there is some evidence that highly urbanized environments favor more innovative individuals [9] and allow higher densities of non-indigenous species ([12]; Sol et al. Unpublished), the hypothesis that in such environments the approach-avoidance conflict interferes less with the innovation process than in other habitats remains to be tested.

To explore the above hypothesis, we exposed mynas from environments with high and low levels of urbanization to three behavioral tests designed to measure neophobia (i.e. aversion to approach novel objects), consumer innovation (i.e. adoption of novel food types) and technical innovation (i.e. exploitation of novel food through new behavioral patterns) [5]. These experimental problem-solving essays sought to imitate key problems that mynas are likely to confront when introduced into novel environments. Our experimental design allowed us to examine differences in innovation ability, neophobic and exploratory behavior as a function of the ecological context from which each myna originated, while controlling for motivation during the problem-solving tests. Furthermore, using path analyses, we explored the most likely causal relationships between habitat, emotional behavior and innovation.

## Materials and Methods

### Ethics statement

All animal care, husbandry, and experimental procedures were in accordance with the Australian code of practice for the care and use of animals for scientific purposes, and were approved by the University of Newcastle Animal Ethics Committee (protocol 1058).

### Study subject

The common myna is naturally distributed throughout south-eastern Asia and has been introduced to Australia, New Zealand, Hawaii, Europe and Mauritius [13]. We trapped 36 adult common mynas in Canberra (Australian Capital Territory, population established in 1960–1970), and 24 in Newcastle (New South Wales, population established in 1970–1975) in two types of habitats: (1) commercial and residential areas dominated by buildings (urban habitat); and (2) suburbs dominated by lawns, shrubs and trees (suburbs). All captures were carried out with a species-specific walk-in baited trap [14] and following the protocol described in detail elsewhere [15], [16], [17], so as to minimize sampling biases in the comparisons across habitats [18]. The trap allows mynas to enter a bottom 1×1×1 m cage through two one-way channels, collect a bait, and then fly up through two additional one-way channels into a 1×1×1 top cage. Birds then rest on perches while consuming the bait. Given the natural tendency of common mynas to aggregate, nearby mynas are attracted by the contact calls of trapped birds and approach more willingly. Birds were transported to Newcastle University by vehicle over short distances in small individual cotton bird bags, or for the 350 km/5 h journey from Canberra in groups of about 10–15 birds in large 1×1×1 m cages equipped with perches and abundant food (dog pellets) and water. Once in the University, we banded individuals with unique color ring combinations, collected standard morphological measures (wing, bill length, tarsus and tail) and placed them in group-aviaries (10–15 individuals from the same habitat and population in each aviary) for at least seven days to acclimate to captivity. Because the common myna is considered a pest in Australia and the government does not allow them to be released once captured, all individuals were euthanized at the end of the experiments via a CO_2_ overdose, using the same procedure as described elsewhere [15], [16]. Sex was determined by post-mortem examination of the reproductive organs, yielding to 17 males and 15 females in the urban habitat and 17 males and 11 females in the suburbs.

### General procedure

The experiments were conducted from June to September, 2007. Each week, we randomly chose either four or six common mynas from the group-aviaries and placed them in indoor individual cages, all containing a nest box, a watering/bathing bowl and a small (4×4×2 cm) feeding dish. Each cohort included individuals from the same population (Canberra or Newcastle), the identity of which was alternated each week. All birds were left for two days to acclimate to their new environment and the experimental sessions took place on each of the following three consecutive days, early in the morning. Birds may gradually habituate to novel stimuli, implying that performance in a novel test may be affected by experience with a novel object in the previous test. Consequently, we chose to conduct the three experimental sessions in a fixed order [19], beginning with the session during which birds underwent the most simple task (i.e., neophobia test) and ending with the session during which birds underwent the most complex task (i.e., technical innovation test).

During experimental sessions, birds were observed from behind a blind to avoid disturbance by the observer. All experiments were videotaped and behavior was scored using Jwatcher [20]. Video-recordings were scored by the same researcher (IB), who was blind with regards to the population of origin of the birds so as to prevent any unconscious biases. Individuals had access ad libitum to food and to water, except overnight when they were food deprived in preparation for morning tests, and during experimental sessions when the experimenter controlled food access. Ad libitum food consisted of dry dog pellets supplemented with chopped fruit and vegetables.

All experimental sessions included a 10-min initial control phase, followed immediately by a 20-min problem-solving phase, which involved the neophobia, the consumer innovation or the technical innovation test. During the initial control phase, the observer waited until the subject had moved away from the usual feeder and then reached his/her hand into the cage through a small hole in the blind and placed two dog pellets in the subject's empty feeding dish. Latency to begin feeding was used as a measure of motivation. Because the birds hid in the nest box every time the experimenter added food or a new test, we estimated the latency to feed from the first time the bird stuck its head out of the box (first visual contact, hereafter). Methodological details for the neophobia, consumer innovation and technical innovation tests are presented next. Two individuals behaved like if they were sick during some of the tests, and hence these individuals were not evaluated.

### Neophobia test

Neophobia, defined as the aversion to approach novel objects [5], was measured using the classical approach of placing an unfamiliar object next to the animal's usual feeding spot [7]. Here, we used a role of yellow tape (5 cm diameter, 2 cm width) and a round green plastic hairbrush of the kind used to brush dog coats (7 cm diameter, 3 cm thick), which are objects that mynas are unlikely to have encountered in the wild. Half the mynas received the yellow tape, while the other half received the green hairbrush.

The neophobia test was initiated immediately after the myna had consumed the food from the initial control phase. The observer waited until the myna had moved away from the feeder, and then reached through the small hole in the blind in order to hang a novel object on a hook next to the bird's feeder and place two dog pellets in the feeder. The performance in the task was measured as the latency from first visual contact to begin eating in presence of the novel object.

### Consumer innovation test

Consumer innovation refers to the acquisition of a novel food using pre-established foraging techniques. To measure consumer innovation in mynas, we used cooked rice, colored either blue or green, as a novel food. The consumer innovation test was initiated immediately after the myna had consumed the food from the initial control phase. The observer waited until the myna moved away from the feeder, and then reached through the small hole in the blind in order to place the novel food in the myna's feeder. To estimate performance in problem solving, we measured the latency from first visual contact to eating the food.

### Technical innovation test

Technical innovation refers to the acquisition of a novel food or a previously used food via the use of a new foraging technique [21]. To measure technical innovation in mynas, we placed two dog pellets in a wooden well (20 mm diameter and 15 mm deep) and covered it with an opaque lid. The lid was fitted with a small (10 mm diameter) metal eye to facilitate manipulation. Following Boogert et al. [22], we habituated individuals to the experimental apparatus (wooden well) prior to the technical innovation test. This was achieved by presenting two dog pellets beside the well during the initial control phase, rather than in the feeding dish, as during all other initial controls. Thus, we reduced the neophobic response to the experimental apparatus per se. The technical innovation test began immediately after the myna had consumed the food from the initial control phase and moved away from it, and was initiated by reaching through the small hole in the blind and putting the dog pellets and lid into place. To estimate performance in problem solving, we used two different measures: (i) the total latency since the first visual contact until the bird opened the well and ate the food; and (ii) the latency since the first bill peck delivered to the wooden well until the bird opened the lid and ate the food. In addition, the number of bill pecks delivered to the wooden well was used as a measure of exploration.

### Analyses

We modeled the problem solving performance in neophobia, consumer innovation and technical innovation tests as a function of the habitat (urban vs. suburban) and a range of other predictors (sex, population, morphology and motivation) using a variety of approaches. The number of bill contacts in the technical innovation test was modeled using a Generalized Linear Model (GLM) with a Poisson error and a log-link using R software (R 2007). In those tests where latency to solve a task was capped at 20 min, statistical modeling was more difficult because not all individuals solved the task. Although in animal behavior these types of variables are usually analyzed with ordinary regressions or ANOVAs, these approaches are inappropriate because censored variables are unlikely to meet the assumption of normality. In addition, regressions and ANOVAs give the same response value to all individuals that failed to complete the task; however, it is quite likely that the individual values would have differed, if individuals had been given more time to solve the task. Survival analyses provide an appropriate alternative framework to analyze censored data when the response variable is latency to solve a task. Specifically, we used the Cox proportional Hazards models, a non-parametric approach that requires few assumptions and allows the inclusion of co-variables in the model [23].

Because of the high correlation between different morphological traits, their inclusion in the models as predictors should have led to problems of co-linearity. To avoid this problem, we used the factors of a Principal Component Analysis (PCA) instead of the actual variables in the analysis. These factors were estimated based on the correlation matrix of the bill, tarsus, tail and wing lengths, all log-transformed (see Table S1). We used the two first factors of the PCA, which together accounted for 89% of variation in morphology.

Finally, we used path analysis to decompose the correlation between innovation propensity and habitat as a function of neophobia and exploration. A path analysis is a multivariate statistical method useful to describe the direct, indirect and spurious dependencies among a set of variables, and it is particularly powerful to identify plausible causal relationships. We built path analyses using AMOS 16.0 [24], fitting general structural equation models using the method of maximum likelihood with multinormal errors. The fit of the models was evaluated with a chi-square test to compare the observed and predicted covariance matrices, as well as by using the Akaike information (AIC) and Bayes information criteria (BIC). The significance of the path coefficients was assessed using survival analysis and GLM, depending on the nature of the endogenous (response) variable, as explained above.

## Results

**Figure 2:**
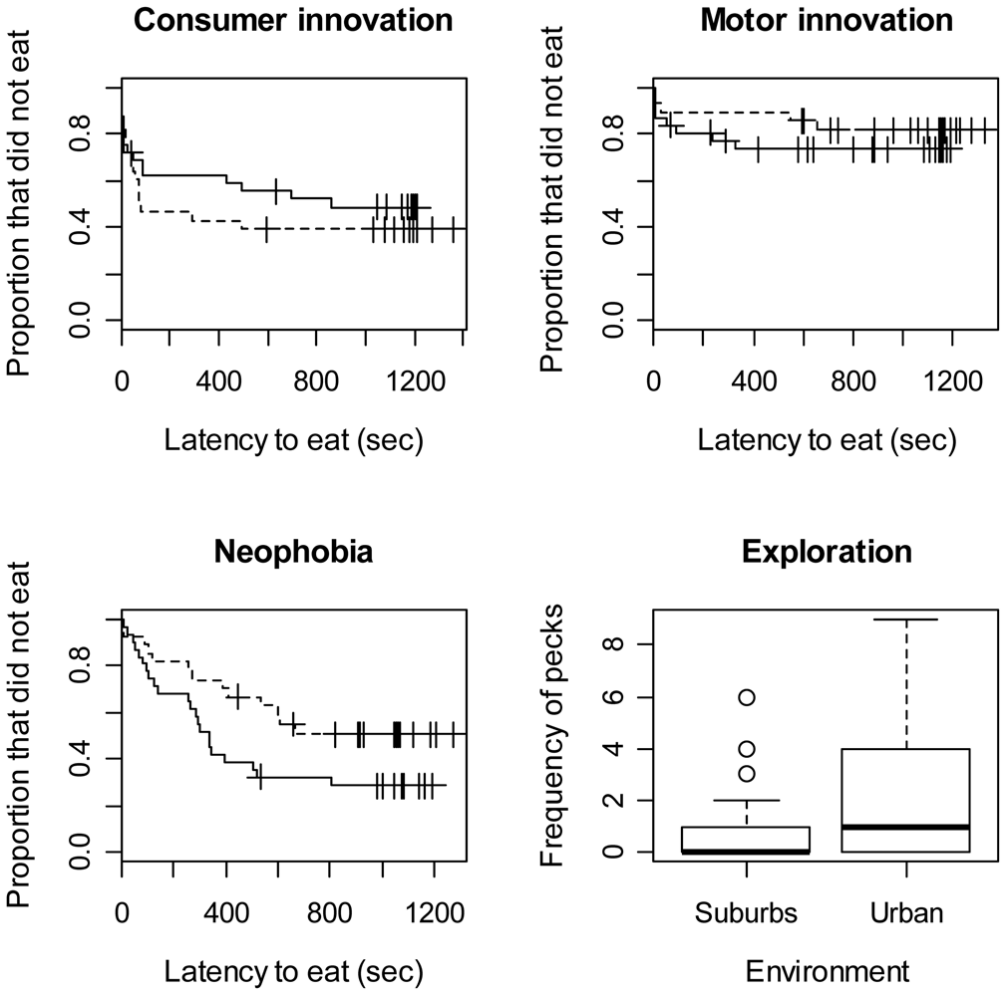
Difference in resource innovation, technical innovation, neophobia and exploration between mynas from the urban and suburbia environments. In the survival curves, solid lines represent birds from the urban environment whereas the dashed lines represent birds from the suburbia.

Given that our main interest was in the innovation process, we started by asking whether mynas from different environments varied in their innovative abilities. For the consumer innovation task, none of the studied variables was significantly associated with latency to solve the task (Fig. 2; Table 1). Nevertheless, for the technical innovation task, individuals that solved the task faster were significantly more likely to originate from the urban habitat (Fig. 2), whether measuring it as the total latency to open the lid and eat the food (Cox model: z = −2.38, P = 0.017) or the latency since the first bill contact with the well (Table 1). The observed association was not caused by differences between individuals in motivation to feed; faster innovators displayed shorter latencies to feed during the initial control phase of the test (Table 1), yet there were no differences in the control latency among habitats (Cox model: z = −0.236, P = 0.814) and consequently motivation did not change the innovation-habitat association (see Table 1). Nor was the association caused by sex or morphological differences between individuals; although faster innovators had higher factor 1 scores in the morphological PCA, indicating disproportionally longer tails, the conclusion that highly urbanized mynas were faster innovators held when morphology and sex were included in the same model (Table 1).

**Table 1:** Survival models relating problem-solving latency in technical innovation, consumer innovation and neophobia as a function of habitat and a set of confounding variables.

*Consumer innovation*					
	coefficient	exp(coef)	S.E. (coef)	z	P
Habitat (suburbs)	0.259	1.296	0.423	0.611	0.5411
Population (Canberra)	0.056	1.057	0.480	0.116	0.9070
Sex (male)	0.435	1.546	0.636	0.684	0.4940
PC1	1.200	3.321	1.728	0.695	0.4871
PC2	2.568	13.035	3.478	0.738	0.4610
Food (green rice)	0.299	1.348	0.372	0.803	0.4223
Motivation	−0.163	0.849	0.157	−1.043	0.2972

*Technical innovation*					
	coefficient	exp(coef)	S.E. (coef)	z	P
Habitat (suburbs)	−3.063	0.047	1.362	−2.248	0.0246*
Population (Canberra)	−0.319	0.727	1.258	−0.253	0.8000
Sex (male)	−1.754	0.173	1.119	−1.568	0.1169
PC1	−14.040	0.000	4.626	−3.035	0.0024*
PC2	0.362	1.437	6.815	0.053	0.9576
Motivation	−0.757	0.469	0.273	−2.773	0.0056*

*Neophobia*					
	coefficient	exp(coef)	S.E. (coef)	z	P
Habitat (suburbs)	−1.160	0.314	0.444	−2.610	0.0091*
Population (Canberra)	−0.099	0.906	0.521	−0.189	0.8500
Sex (male)	−0.250	0.779	0.640	−0.390	0.6962
PC1	−5.474	0.004	1.863	−2.938	0.0033*
PC2	0.328	1.389	3.046	0.108	0.9141
Object (yellow tape)	0.078	1.081	0.372	0.210	0.8336
Motivation	−0.430	0.651	0.148	−2.897	0.0038*

In categorical variables (habitat, population, sex, food color and type of object), the reference category was set to zero and compared with the category shown between brackets. Variables that were retained in the minimum adequate model are indicated with an asterisk.

**Figure 3:**
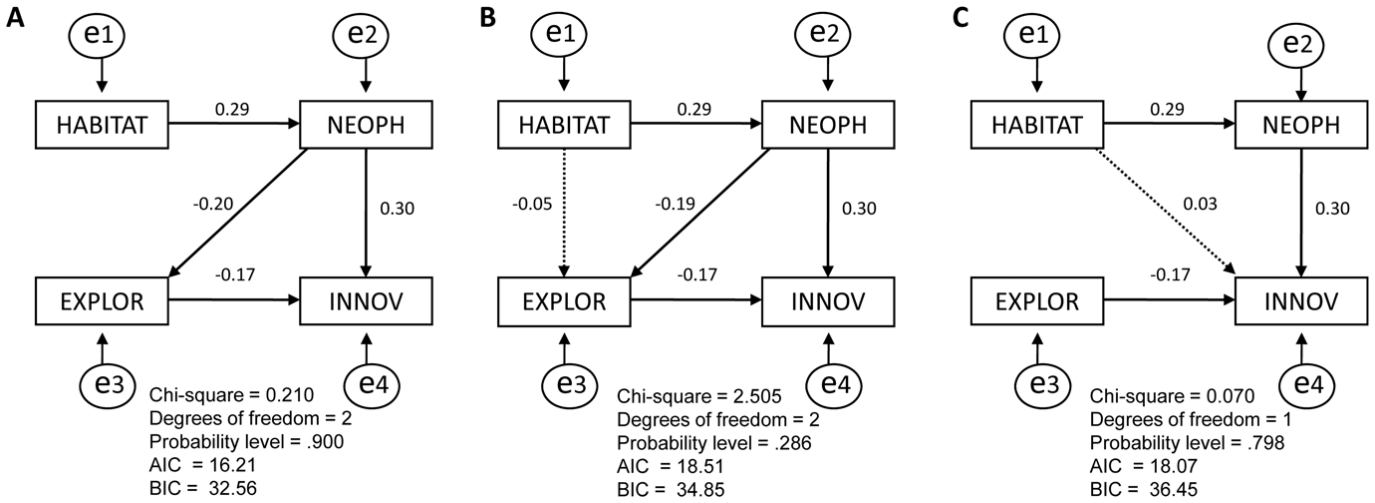
Path models (A–C) deconstructing direct and indirect effects in the relationship between technical innovation propensity (INNOV) and habitat (HABITAT) as a function of neophobia (NEOPH) and exploration (EXPLOR). Solid lines indicate the paths that are significant at *P*<0.05. All models fit well to the data, as indicated by the non-significance of the Chi-square tests, yet model A performs better than the others based on its lower values of AIC and BIC and the significance of all the paths. The terms e1–e4 refer to the error terms.

Having shown that individuals from different environments varied in their innovative abilities, we then asked to what extent differences in the way they weight approach over avoidance underpins differences in innovation. Latency to feed in the presence of a novel object was shorter for urban individuals than it was for suburban birds, suggesting lower neophobic responses, even when other confounds were included in the same model (Fig. 2; Table 1). In addition, mynas from urban habitats showed significantly greater pecking frequencies in the technical innovation experiment than those from the suburbs once the effect of all other confounding variables was taken into account, indicating that they were more exploratory (Table 2, Fig. 2; after removing an outlier: z = −4.23, P<0.0001). It is thus conceivable that variation in technical innovation performance in mynas reflects differences in the way birds from different habitats prioritize approach over avoidance, rather than differences in creativity. We analyzed this possibility with a path analysis. The best model (Fig. 3A) suggests that habitats differences in latency to solve the technical innovation task were caused by the effect of neophobia, which affected innovation directly but also indirectly by reducing physical exploration of the apparatus.

**Table 2:** Poisson GLM relating pecking frequency in technical innovation as a function of habitat and a set of confounding variables.

	coefficient	S.E.	z	P
Habitat (urban)	0.954	0.269	3.55	0.0004*
Population (Newcastle)	−0.315	0.310	−1.01	0.3103
Sex (male)	−0.061	0.291	−0.21	0.8337
PC1	−3.907	1.025	−3.81	0.0001*
PC2	1.240	1.779	0.70	0.4856
Motivation	5.620	6.883	0.82	<0.0001*

In categorical variables (habitat, population and sex), the reference category was set to zero and compared with the category shown between brackets. Variables that were retained in the minimum adequate model are indicated with an asterisk.

## Discussion

The propensity for behavioral innovation has been identified as an important feature of many successful invaders [3], [4]. The challenges that one species must confront when exposed to a new environment are diverse, and include the discovery and adoption of novel resources and the avoidance of previously unknown enemies. Thus, the construction of novel behaviors, whether it be to consume novel foods or to produce novel motor patterns, should facilitate establishment in novel environments. Such feeding innovations may be especially advantageous in urbanized environments, where a wide variety of human-derived resources represent a substantial part of the animals's diet and food access may be hindered by the presence of packaging [8].

Consistent with these considerations, we found that mynas originating from highly urbanized environments showed a higher propensity to innovate than those from less urbanized environments. These differences could not be explained by biases in the sex or age of individuals, or by differences between individuals in morphology. Likewise, Liker & Bókony [9] reported that urban house sparrows, *Passer domesticus*, were more successful in opening a familiar feeder in an unfamiliar way than rural sparrows, and Møller [10] showed that bird species that lived in highly urbanized habitats were characterized by high rates of feeding innovation (but see Kark et al. [11]). Thus, evidence is accumulating that highly urbanized birds are more innovative than less urbanized birds. The question is then, why?

The tendency of highly urbanized mynas to be better problem solvers could indicate that they are more ‘creative’ than less urbanized ones [25]. However, the finding that highly urbanized individuals were faster in solving the motor innovation task, but not the consumer innovation task, does not support for the existence of innovative ‘personalities’ in urbanized common mynas. Moreover, the path analyses suggest that the effect of habitat on latency to innovate was not direct, but indirectly caused by the effect of other behavioral traits (see below).

Motivational state is another feature known to influence innovation propensity [25], [26]. In cities, where the density of some species may be very high ([8]; see Text S1), competition for food may be more intense and thus birds may be hungrier and more motivated to feed. We controlled for this in two ways. First, all mynas were fed ad libitum during the acclimatization period, which contributed to equalize their body condition. Second, all experimental sessions included an initial control phase, in which we quantified the latency to begin feeding in the familiar feeder after overnight food deprivation. This measure should in part reflect how hungry individuals were, and may thus we used as a measure of motivation [19]. The latency to feed during the initial control did not differ among mynas from different habitats, and hence habitat remained associated with latency to solve the technical innovation task when differences in the initial control latency were statistically controlled for.

On the contrary, the emotional response of mynas to novelty, which is known to play a decisive role in the problem-solving ability in other bird species [19], [ but see 27], provides a strong basis for understanding why highly urbanized birds are more innovative than less urbanized birds. Neophobia varied depending on the type of habitat the myna lived in: mynas from highly urbanized environments were significantly less neophobic than mynas from less urbanized habitats. One possible explanation for such differences is that these environments vary in the level of predation risk they impose on individuals. Mynas respond particularly strongly to raptors and learn readily about novel aerial threats [15], [17]. In our study region, raptors are absent from highly urbanized areas, but can be sited in suburban environments that lie adjacent to bushland and rural landscapes (see Text S1). Parallel observations in free-ranging mynas revealed that mynas that inhabit more urbanized environments display shorter flight initiation distances, indicative of reduced predation risk perception. Thus, it is quite possible that a reduced predation pressure leads individuals to favor approach over avoidance when confronted with a conflict between the two behaviors, thus paving the way for innovation. In addition to being less neophobic, mynas from urban habitats tended to peck the experimental apparatus more frequently than mynas from the suburbs. Because the probability of solving the technical innovation task increases with the number of pecks (Sol et al, unpublished), this higher pecking frequency of highly urbanized mynas is likely to reflect a higher propensity for exploration. Interestingly, our path analyses suggest that habitats differences in latency to solve the technical innovation task were primarily caused by the effect of neophobia, which affected innovation directly but also indirectly by reducing physical exploration of the apparatus. This finding points to one possible explanation for the finding that latency to adopt novel foods did not vary among habitats, and that is that neophobia and exploration play a lesser role in consumer innovation than in technical innovation.

Besides varying with the ecological characteristics of the habitat, the propensity to innovate has also been shown to vary over the course of the invasion process. In experiments with house sparrows, the latency to consume novel foods and avoid novel objects was shorter for an invading population than it was for an established population [28], providing evidence that behavioral innovation may be an important mechanism mediating invasion. In our case, mynas were collected from two different geographical regions; however, because mynas were introduced to these areas at approximately the same time (1970s), one would not have expected any behavioral differences. Accordingly, we found no effect of population on performance. Further, history says that mynas were first introduced to the highly urbanized areas, where they are still more abundant, and then dispersed to the suburbs [29]. Thus, individuals from the suburbs could be regarded as more recent invaders than urban ones. Yet, despite this, their latencies to adopt a novel food and avoid feeding close to novel objects were longer, not shorter. This suggests that our results are not confounded by invasion stage.

Overall, our results fit well with the hypothesis that mynas modulate their problem-solving ability according to both the benefits and the risks of innovating in their particular habitat. From an invasion biology perspective, our finding that in highly urbanized environments the need to adopt new foods does not strongly conflict with the hesitation to take risks during exploration is important because it offers an explanation for why many avian invaders reach their highest densities in such environments [12] (see Text S1). In addition, the minor role that neophobia played in consumer innovation suggests that the approach-avoidance conflict interferes little with the incorporation of novel foods in the individual's repertoire. For an invader, a short latency to explore and taste novel foods may be critical to survive in a novel environment because it is likely that many of the new food opportunities it encounters may be exploited with no need of modifying motor patterns [see 30].

Although urban environments may indirectly favor more innovative individuals, it remains to be determined to what extent the observed differences reflect plastic behavioral adjustments or evolutionarily selected genetic change. Evidence from other species indicate that neophobic responses and exploration tendencies may be inherited [7], [31], so favoring approach over avoidance may reflect the expression of a stable temperament trait that is selected for under particular ecological circumstances. Nevertheless, mynas readily learn about their environment, both through their own experience and from watching conspecifics [15], [16], [17], [32]. Consequently, it is also quite possible that the two main drivers to innovation found here –neophobia and exploration- are readily adjusted based on experience to suit prevailing ecological conditions. Future work should have to elucidate whether the observed divergence in neophobia and exploration, and hence in the propensity of individuals to innovate, are the consequence of natural selection or plastic behavioral adjustments.

## Supporting Information

Figure S1 **Variation in (A) abundance of mynas (individuals recorded per transect), (B) latency to exploit novel foraging opportunities (log-transformed), (C) flight distance (log-transformed) and (D) abundance of raptors (individuals recorded per transect) with different degree of urbanization.**
(DOC)

Table S1 **Loadings of the PCA on morphological variables.**
(DOC)

Text S1 **Field tests of the assumptions.**
(DOC)
