# Supplementary material for: Exploring or Avoiding Novel Food Resources? The Novelty Conflict in an Invasive Bird
Source: PLoS One. 2011 May 18;6(5):e19535. doi: 10.1371/journal.pone.0019535 (PMC3097186; doi:10.1371/journal.pone.0019535)
Supplement: Figure S1 — Variation in (A) abundance of mynas (individuals recorded per transect), (B) latency to exploit novel foraging opportunities (log-transformed), (C) flight distance (log-transformed) and (D) abundance of raptors (individuals recorded per transect) with different degree of urbanization. (DOC) [file pone.0019535.s001.doc]

Supplementary figure S1
 Figure S1. Variation in (A) abundance of mynas (individuals recorded per transect), (B) latency to exploit novel foraging opportunities (log-transformed), (C) flight distance (log-transformed) and (D) abundance of raptors (individuals recorded per transect) with different degree of urbanization.
