# Supplementary material for: Exploring or Avoiding Novel Food Resources? The Novelty Conflict in an Invasive Bird
Source: PLoS One. 2011 May 18;6(5):e19535. doi: 10.1371/journal.pone.0019535 (PMC3097186; doi:10.1371/journal.pone.0019535)
Supplement: Table S1 — Loadings of the PCA on morphological variables. (DOC) [file pone.0019535.s002.doc]

**Supporting Information**

**Exploring or avoiding novel food resources? The novelty conflict in an invasive bird**

Daniel Sol^1,2*^, Andrea S. Griffin^3^, Ignasi Bartomeus^1^ and Hayley Boyce^3^

^1^Centre for Ecological Research and Forestry Applications (CREAF) Autonomous university of Barcelona, 08193 Bellaterra, Catalonia, Spain

^2^Center for advanced Studies of Blanes (CEAB), Spanish National Research Council (CSIC)

^3^School of Psychology, University of Newcastle, Callaghan, NSW 2308
Australia

**Supplementary table S1**

Table S1: Loadings of the PCA on morphological variables.

| Variable | Comp. 1 | Comp. 2 | Comp. 3 | Comp. 4 |
| --- | --- | --- | --- | --- |
| Bill | - | 0.919 | 0.39 | - |
| Tarsus | - | 0.351 | -0.771 | -0.523 |
| Tail | -0.989 | - | 0.141 | - |
| Wing | - | 0.182 | -0.483 | 0.851 |
| Proportion of Variance | 0.656 | 0.229 | 0.097 | 0.017 |
