# Supplementary material for: Exploring or Avoiding Novel Food Resources? The Novelty Conflict in an Invasive Bird
Source: PLoS One. 2011 May 18;6(5):e19535. doi: 10.1371/journal.pone.0019535 (PMC3097186; doi:10.1371/journal.pone.0019535)
Supplement: Text S1 — Field tests of the assumptions. (DOC) [file pone.0019535.s003.doc]

**Supplementary text S1**

*Field tests of the assumptions*

In parallel with the experiments, we carried out observations and experiments in the field to validate the assumptions of our study. Field work was carried out from June to August 2007 in a study area centred in Newcastle, a city on the eastern coast of Australia, with the limits being Sydney to the south, Nelson bay to the North, and the Blue mountains to the interior (see Sol et al., submitted). All observations and experiments were carried out by one of us (D. S.).

We first validated the assumption that common mynas attain higher success in the most urbanized environments by estimating the abundance of the species along gradients of urbanization. We identified twenty-four gradients of urbanization at least 500m apart, each one comprising three habitats: (1) commercial and residential areas dominated by buildings (urban habitat); (2) suburbs dominated by lawns, shrubs and trees (suburbs); and (3) bush fragments where native vegetation predominated (wildland). We recorded the number of birds observed in transects well within each habitat type during 20-min within a 50 m belt. Counts in each gradient were carried out on the same day, either in the morning or the evening, taking care to randomize the order in which each habitat was prospected. Modelling abundance with a generalized linear mixed model [1] with Poisson error and gradient as random factor, we found that common mynas were more abundant in the urban habitat than in both the suburbs and in the wildland (t = -5.38, df = 35, P < 0.001; Fig. S1A).

The second assumption we sought to validate using a field experiment was that of whether highly urbanized mynas are greater foraging opportunists than less urbanized mynas. In both urban and suburban environments, we located a foraging bird and dropped small pieces of bread to attract it. We used bread because this is one of the food resources more easily found in urban environments. We noted whether the individual responded or not to the food opportunity. If it responded, we recorded the time until a piece of bread was handled. The experiment was capped after 3-min. To control for motivation and other confounds, we noted the time of day and group size for each experiment. We carried out 59 tests in different individuals, 37 from the urban habitat and 22 from the suburbs. A survival analysis revealed that mynas from urban habitats showed shorter latencies to exploit the new feeding opportunity than those from the suburbs (Cox model: z = 4.62, P < 0.0001; Fig. S1B).

The last assumption we validated is whether highly urbanized mynas have and perceive fewer risks than less urbanized mynas. To validate the perceived risk, we measured initiation flight distances of foraging mynas in response to an approaching human [2,3,4]. Flight distances are thought to reflect the risks that an individual is willing to take, reflecting the tradeoff between the benefits acquired by current activity and the costs of fleeing during the approach of a potential predator [2]. At a mechanism level, we expect that in cities the increased exposure to potential predators that never eat you (i.e. humans) produces habituation to predators, decreasing the flight distance. Yet this will reflects that individuals live in an environment where the risk of being eaten by a predator is low. When an individual was located foraging on the ground, the observer moved at a normal walking speed towards the individual and recorded the distance at which the individual took flight with a meter. We also noted the location, time of day and group size for each test. We obtained 80 measures of flight distance, 63 in urban environments and 17 in the suburbs. The analysis supports the assumption that highly urbanized mynas show shorter flight distances than less urbanized mynas (Least-square regression: t = 4.19, P < 0.001; Fig. S1C). The real risk was quantified by counting the number of raptors observed along the 24 transects. We observed eight raptors during the 24 transects, seven in the suburbs and only one in the urban environment (Fig. S1D).

**References**

1. Blackburn TM, Duncan RP (2001) Determinants of establishment success in introduced birds. Nature 414: 195-197.

2. Blumstein DT (2006) Developing an evolutionary ecology of fear: how life history and natural history traits affect disturbance tolerance in birds. Anim Behav 71: 389–399.

3. Fernández-Juricic E, Jimenez MD, Lucas E (2001) Bird tolerance to human disturbance in urban parks of Madrid, Spain. Management Implications. In: Marzluff JM, Bowman R, Donnelly R, editors. Avian Ecology and Conservation in an Urbanizing World. Norwell, MA: Kluwer Academic. pp. 261-275.

4. Møller AP (2009) Successful city dwellers: a comparative study of the ecological characteristics of urban birds in the Western Palearctic. Oecologia 159: 849-858.
